# Supplementary figures and images for: Simple indices of infarct size post ST-Elevation Myocardial Infarction (STEMI) provides similar risk stratification to cardiac MRI
Source: PLoS One. 2024 Nov 21;19(11):e0311157. doi: 10.1371/journal.pone.0311157 (PMC11581229; doi:10.1371/journal.pone.0311157)

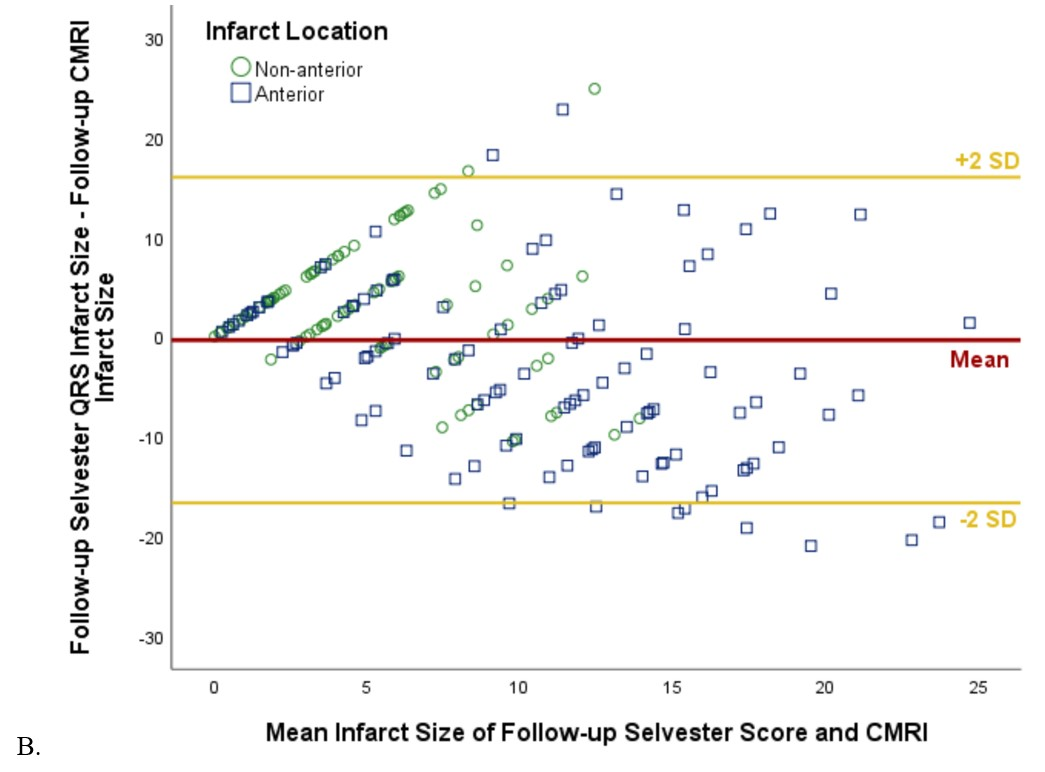

Supplement: S1 Fig — (ZIP) [file pone.0311157.s001.zip › S1B_Fig.tif]

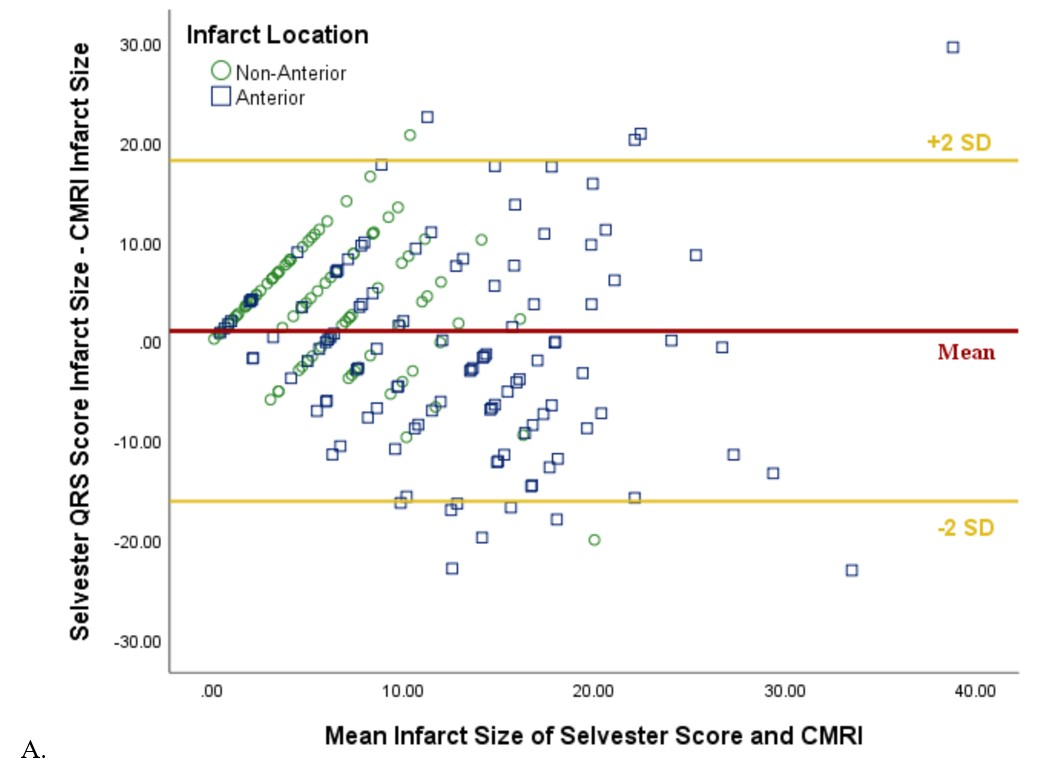

Supplement: S1 Fig — (ZIP) [file pone.0311157.s001.zip › S1A_Fig.tif]
